# Supplementary material for: The interactome of intact mitochondria by cross-linking mass spectrometry provides evidence for coexisting respiratory supercomplexes
Source: Mol Cell Proteomics. 2017 Dec 8;17(2):216–32. doi: 10.1074/mcp.RA117.000470 (PMC5795388; doi:10.1074/mcp.RA117.000470)
Supplement: Supplemental Data [file supp_RA117.000470_133922_0_supp_23978_fzffwf.zip › spectra_annotation/mito_DR_spectra_annotation/309-1-5-1-3-1.pdf]

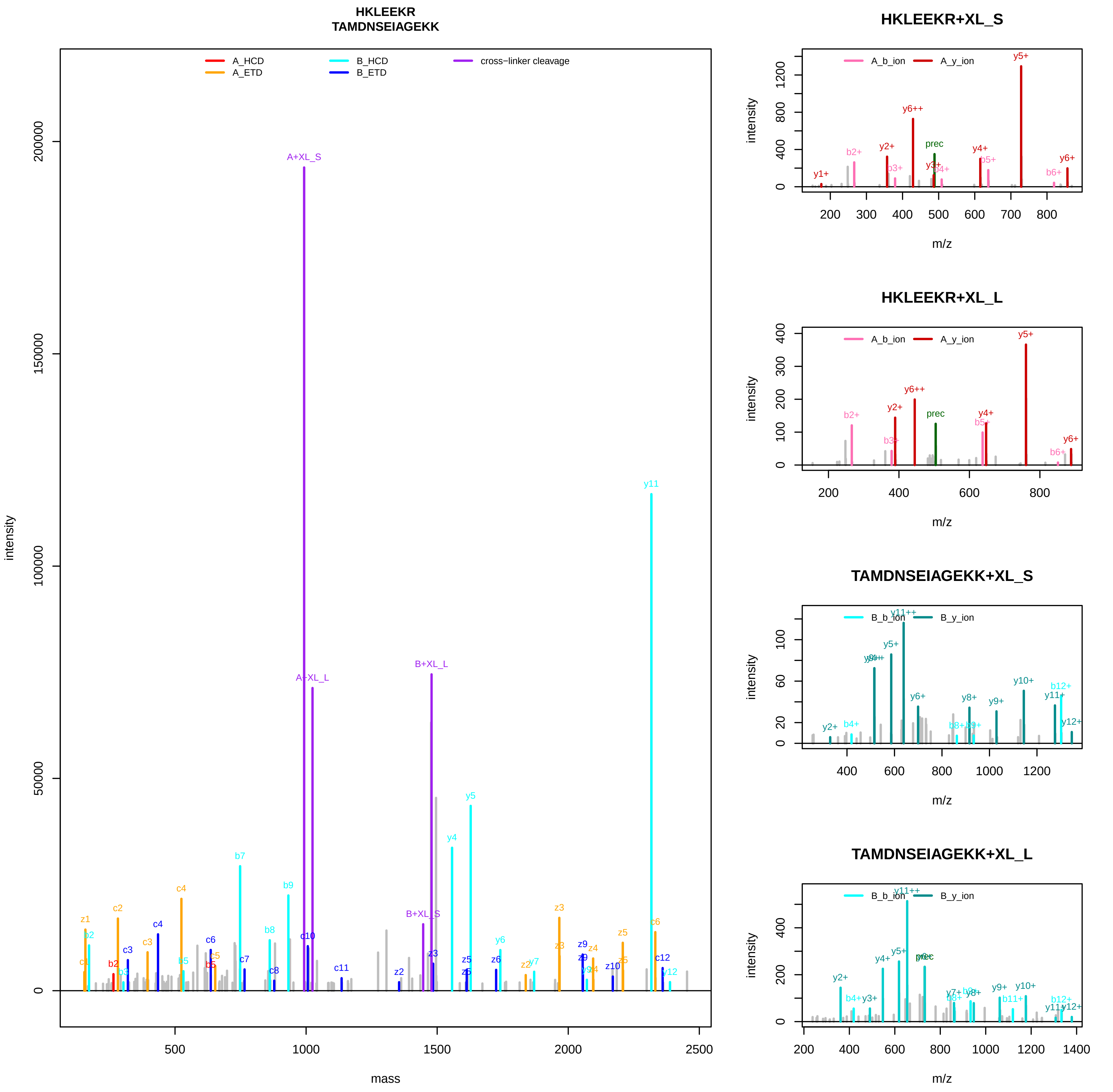

HKLEEKR
TAMDNSEIAGEKK
A_HCD
A_ETD
B_HCD
B_ETD
cross−linker cleavage
A+XL_S
A+XL_L
B+XL_L
B+XL_S
y11
y5
y4
b7
b9
b8
c4
c2
z1
c10
c6
z3
z5
intensity
mass
HKLEEKR+XL_S
A_b_ion
A_y_ion
y5+
y6++
prec
y2+
b2+
y4+
b5+
y6+
b6+
y1+
m/z
HKLEEKR+XL_L
TAMDNSEIAGEKK+XL_S
B_b_ion
B_y_ion
y11++
y5+
y6+
y8+
y9+
y10+
b12+
y11+
y12+
y2+
b4+
TAMDNSEIAGEKK+XL_L
y4+
y9+
y10+
